# Biochemical and nutritional profile of maize bran‐enriched flour in relation to its end‐use quality

**DOI:** 10.1002/fsn3.2323

**Published:** 2021-05-04

**Authors:** Muzzamal Hussain, Farhan Saeed, Bushra Niaz, Muhammad Afzaal, Ali Ikram, Shahzad Hussain, Abdellatif A. Mohamed, Mohamed S. Alamri, Faqir M. Anjum

**Affiliations:** ^1^ Department of Food Science Government College University Faisalabad Pakistan; ^2^ Department of Food Science & Nutrition King Saud University Riyadh Riyadh Saudi Arabia; ^3^ University of the Gambia Serrekunda Gambia

**Keywords:** bread quality, maize bran‐enriched flour, nutritional composition, rheological properties, water‐holding capacity

## Abstract

The core objective of current research was determined to nutritional and bioactive profile of maize bran (MB)‐enriched flour in relation to its end‐use product quality. Furthermore, rheological properties of MB‐enriched flour at different levels (5%, 10%, and 15%) were explicated through farinograph and mixograph. Moreover, bread was prepared with the addition of MB‐enriched flour and was characterized for nutritional and textural properties. Results showed that MB‐enriched flour having high water absorption and water retaining potential up to 4%–7% as compared to wheat flour (WF). Moreover, dough height gradually decreased with the addition of MB due to water‐binding ability of bran which causes a decrease in gas retention during fermentation. This resulted in bread volume decrease (4%–7%) as compared to WF. Furthermore, the moisture content and hardness increased with the addition of MB. The water activity of bread slightly increased with the addition of maize bran after 4‐day storage. Conclusively, MB‐enriched flour improved nutritional, textural, and sensorial properties of final product.

## Graphical abstract

Maize bran‐enriched flour is rich source of nutritional and bioactive components. Maize bran‐enriched flour bread has high dietary fibers than wheat flour bread. MB‐enriched flour bread explicated high score of sensorial overall acceptability.

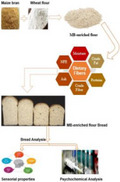

## 1 INTRODUCTION

Consumers are now demanding foods that have not only appetizing smell, taste, and appearance but also healthy ingredients. Cereal brans are generally used in various foods to improve their nutritional profile with special reference to dietary fibers (Han et al., 2019). In recent years, interest in dietary fiber has been improved owing to its health benefits such as diabetes, coronary heart disease, hypertension, stroke, and cancer (Özkaya et al., 2018).

Maize (*Zea mays*) is a major crop among cereals all over the world. During maize processing, the milling process produces 60–70 g/kg of maize bran (Zhao et al., 2014). Maize bran is a milling item produced from the hard‐outer corn kernel layer. It is very high in fiber like the other cereal brans and widely used in different food products (Herrera‐Balandrano et al., 2020). However, maize bran has less value and is frequently used alone or in blend with maize germ cake or meal for animal feed (Zhao et al., 2014). Maize bran comprises approximately heteroxylans 50%, cellulose 20%, phenolic acids (ferulic and diferulic acid) 4%–5%, proteins 10%–12%, starch 4%–5%, lipids 3%–4%, and ash 2% (Carvajal‐Millan et al., 2007). Many food industries utilized this substance for lowering the caloric value of snack foods, and as filler in their food items. It is used in different food products to improve fiber content of final product (Afangide et al., 2018).

Among different cereal products, bread has much importance and is usually prepared from wheat flour, sugar, yeast, water, salt, and fat (Okafor et al., 2012). Bread is primarily prepared with wheat flour, but it has low mineral and dietary fiber as well as low vitamins and proteins (Lu et al., 2018). With the supplementation of nutrients, it becomes an excellent food product that will ultimately improve nutritional condition of consumers (Alamu et al., 2018). Arabinoxylans (Saeed et al., 2016), alhydwan seeds (Ammar et al., 2016), dietary fiber (Packkia‐Doss et al., 2019), Pumpkin seed flour (Agu et al., 2010), banana, aonla and sapota powder (Rajeswari et al., 2018), and protein concentrates (Alzuwaid et al., 2020) have been incorporated into bread to increase its quality and nutritional composition. Boita et al. (2016) described the rheological properties of wheat flour dough and bread with the addition of wheat bran. Fortifying staple foods such as bread and maize bran are a particularly accessible and economical source of dietary fiber, protein, and phytochemicals, as its excellent insoluble dietary fiber content, and antioxidants including ferulic acid, diferulic acid, and p‐coumaric acid (Bento‐Silva et al., 2018).

However, the bran addition in bread contributes to practical and functional improvements in the cycle of bread making and organoleptic properties including crumb softness and reduction in bread loaf volume (Hemdane et al., 2018). In addition to diluting gluten when bran is incorporated in bread, the maize bran properties play a crucial role in potential interactions between bran and flour components (Hemdane et al., 2016). It has several functions like enhancing water absorption, lowering tolerance for dough strength, mixing, fermentation, and dough stickiness improved, when maize bran is incorporated into the flour. Thus, the incorporation of cereal bran leads to lower loaf volume, crumb softness, specific volume, and causes darker crumb color and coarser crumb texture (Özkaya et al., 2018).

Hypothesis: Maize bran may or may not have positive impact on biochemical, nutritional and rheological characteristics of wheat flour. Objective: In this research work, the effects of maize bran on biochemical composition, water‐holding capacity, farinographic and mixographic characteristics of MB‐enriched flour were determined. In the end, bread was prepared with the addition of different levels of MB and was analyzed for its textural and sensorial characteristics.

## 2 MATERIALS AND METHODS

The research work was carried out at the Department of Food Sciences, Government College University Faisalabad. A rheological study was conducted at Wheat Research Institute, Ayub Agricultural Research Institute (AARI), Faisalabad. Maize bran (Buffalo MB 135000) was procured from Rafhan Maize Product Co Ltd. Faisalabad, Pakistan. Maize bran was commercially micronized (particle size <250 µm) by using milling process.

### 2.1 Addition of maize bran in wheat flour

Maize bran (MB) was added with the different proportions of 0% control (WF), 5% (MB5), 10% (MB10), and 15% (MB15) in wheat flour to develop maize bran‐enriched flour.

### 2.2 Nutritional composition of MB‐enriched flour

MB‐enriched flour treatment samples were evaluated for proximate composition including moisture content of flour determined by method no. (44–15.02), crude fat content evaluated by method no. (30–10.01), crude protein determined by the percentage of nitrogen in flour method no. (46–19.01), basic ash content was determined by using method no. (08–01.01), crude fiber content was determined by using method no. (32–10.01), described in AACC (2000).

### 2.3 Total dietary fiber of MB‐enriched flour

Total dietary fiber, soluble, and insoluble dietary fibers of all the treatment groups WF, MB5, MB10, and MB15 were analyzed by the following method no. 32‐05, AACC (2000).

### 2.4 Water‐holding capacity of MB‐enriched flour

Water‐holding capacity (WHC) of wheat flour (WF) and bran‐enriched flour (MB5, MB10, and MB15) was measured according to Hemdane et al. (2018) protocol. 1 g sample was weighed in a 50 ml centrifuge tube and 10 ml distilled water was added to the solution. Then, the sample was stirred by using a vortex mixer and left for 40 min at room temperature and centrifuged at 10,000 RPM for 10 min. The supernatant was gently separated, and a drainage process of 15 min was done to remove excess water that was not retained. Weighed the centrifuge tube, and declared the WHC as g water retained per g of dry matter.

### 2.5 Farinographic Analysis of MB‐enriched flour

The farinographic characteristics including water absorption capacity, dough development time, dough stability, mixing tolerance index, and dough softness of dough obtained from various blends of wheat flour with maize bran treatments (WF, MB5, MB10, and MB15) were analyzed through farinograph by following method no. 54‐21.01 of AACC (2000).

### 2.6 Mixographic characteristics

Mixographic properties such as mixing time and peak height of the dough obtained from various blends of wheat flour with maize bran treatments (WF, MB5, MB10, and MB15) were analyzed through mixograph by following method no. 54‐40.02 of AACC (2000).

### 2.7 Preparation of bread

The ingredients, MB‐enriched flour, milk powder, compressed fresh yeast, sugar, salt, improver, shortening, and water were mixed with mixer and prepared bread with straight dough bread baking method no. 10‐10.03 (AACC, 2000). Comparison of dough height after molding and bread height after baking was measured to estimate fermentation effects on MB samples.

### 2.8 Moisture content

The moisture content of fresh‐baked bread was measured by using a hot air oven at 105 ± 5°C temperature by following the AACC (2000) method no. 44‐15.02.

### 2.9 Textural analysis of bread

The hardness of the bread was measured by some modification of the bread compression test described by Saeed et al. (2016). In this method, the sample of bread was compressed twice by up to 50%. Deformation and rehabilitation behavior under stress demonstrated crumbs of firmness.

The fracturability of bread was evaluated by a cylindrical ball die that penetrates the bread slices up to 40% followed by method no. 74‐09.02 (AACC, 2000).

### 2.10 Bread loaf volume

After baking, the bread was cool down for 15 min, and the loaf volume was estimated by the rapeseed replacement method by following the AACC method 10‐05.01 (AACC, 2000).

### 2.11 Water activity of bread

The water activity of bread samples was indicated by using the Hygropalm Water Behavior Meter (Retronic, Rotronic Instrument Corp., UK) followed by the method illustrated by Piga et al. (2005).

### 2.12 Sensory characteristics of bread

Bread treatments WF, MB5, MB10, and MB15 were prepared for sensory evaluation. Sensory acceptance of bread was tested by twenty panelists (Ph.D. scholars and some faculty members of Department of Food Sciences Government College University Faisalabad, Pakistan). Each panelist was presented with four coded samples in a sensory booth and asked to evaluate for appearance, color, taste, flavor, texture, and overall acceptability on a 9‐point hedonic scale, where 1 was disliked extremely and 9 was like extremely. Statistical analysis of data was carried out using SPSS statistics 21. The *F* test value was obtained, and a multiple comparison test was performed on the means, using Duncan's Multiple Range Test at 0.05 levels.

### 2.13 Statistical analysis

All experiments were carried out in three replicates. Analysis of variance (ANOVA) was conducted by using the SPSS statistics 21. The *F* test value was obtained, and a multiple comparison test was performed on the means, using Duncan's Multiple Range Test at 0.05 levels.

## 3 RESULTS AND DISCUSSION

### 3.1 Nutritional composition of MB‐enriched flour

The flour samples were analyzed for moisture content, crude protein, crude fat, crude fiber, NFE, and ash content. The nutritional composition of different flour samples and their mean values are mentioned in Table 1.

**TABLE 1:** Chemical composition of composite flour treatments

Flour Treatments	Moisture (%)	Ash (%)	Crude protein (%)	Crude Fat (%)	Crude Fiber (%)	NFE (%)	TDF (%)	SDF (%)	IDF (%)
WF	12.48 ± 0.04^b^	0.35 ± 0.01^a^	7.98 ± 0.03^a^	1.13 ± 0.02^a^	2.01 ± 0.02^a^	73.33 ± 0.05^c^	3.38 ± 0.03^a^	1.03 ± 0.02^a^	2.35 ± 0.01^a^
MB5	12.32 ± 0.09^c^	0.66 ± 0.02^a^	8.17 ± 0.01^a^	2.07 ± 0.03^b^	2.18 ± 0.02^a^	72.51 ± 0.13^c^	8.81 ± 0.04^a^	1.12 ± 0.01^a^	7.69 ± 0.03^b^
MB10	12.16 ± 0.05^b^	0.78 ± 0.02^a^	8.32 ± 0.06^b^	2.87 ± 0.01^b^	2.37 ± 0.01^b^	71.81 ± 0.06^b^	12.52 ± 0.09^b^	1.31 ± 0.04^b^	11.21 ± 0.05^b^
MB15	11.69 ± 0.01^a^	1.02 ± 0.05^b^	8.54 ± 0.06^c^	3.12 ± 0.02^c^	2.98 ± 0.03^b^	70.95 ± 0.04^a^	15.41 ± 0.05^c^	1.32 ± 0.05^b^	14.09 ± 0.07^c^

Abbreviations: IDF, Insoluble dietary fiber; MB10, 10% maize bran + 90% wheat flour; MB15, 15% maize bran + 85% wheat flour; MB5, 5% maize bran + 95% wheat flour; NFE, Nitrogen‐free extract; SDF, Soluble dietary fiber; TDF, Total dietary fiber; WF, Wheat flour.

Moisture content is important aspect to control physiochemical properties of flour. Moisture is important in determining the shelf life of the flour. The mean value of moisture content was 12.48 ± 0.04 in WF (control) sample. The moisture content in MB5, MB10, and MB15 treatments was 12.32%, 12.16%, and 11.69%, respectively. According to Turkish Food Codex (1999) and Codex Alimentarius standards, maximum moisture should be 14.5% and 15.5%, respectively. When the moisture content of flour rises above 14%, it is more susceptible to fungal growth, flavor change, and enzymatic activity (Batool et al., 2012).

The ash content of the flour represents the inorganic residues after the combustion of organic material which contains a small amount of minerals. Elawad et al. (2016) explored that the ash content of cereal/legume bran composite flour ranged between 0.8% and 2.3%. The mean value results of Ash content in WF, MB5, MB10, and MB15 were 0.35 ± 0.01%, 0.66 ± 0.02%, 0.78 ± 0.02%, and 1.02 ± 0.05%, respectively.

Crude protein is estimated after measuring the nitrogen content of a food. In the case of present research work, the protein contents of all samples were 7.98 ± 0.03% (WF), 8.17 ± 0.01% (MB5), 8.32 ± 0.06% (MB10), and 8.54 ± 0.06% (MB15). In all treatments, the average protein content ranges meet the standards (minimum 7.0%) given by Codex Standard for wheat flour (Codex Stan 152‐ 1995). The fat content was 1.13 ± 0.02, 2.07 ± 0.03, 2.87 ± 0.01, and 3.12 ± 0.02 in flour treatments WF, MB5, MB10, and MB15 sample, respectively.

The crude fiber content can be related to grain size and bran portion thickness in grain. The content of crude fiber is positively associated with the level of bran in the flour (Gajula, 2017). The crude fiber content in WF, MB5, MB10, and MB15 was 2.01 ± 0.02, 2.18 ± 0.02, 2.37 ± 0.01, and 2.98 ± 0.03%, respectively. The bran has high crude fiber content; therefore, bran addition in flour causes high crude fiber content in flour. The NFE content in WF, MB10 and MB15 was (73.33 ± 0.05%), (72.51 ± 0.13%), (71.81 ± 0.06%) and (70.95 ± 0.04%), respectively. Elawad et al. (2016) reported that wheat bran (ratio 4%) composite flour contains 69.6% NFE.

### 3.2 Total Dietary Fiber (soluble and insoluble dietary fiber)

The total dietary fiber content in flour treatments WF, MB5, MB10, and MB15 was 3.38 ± 0.03, 8.81 ± 0.04, 12.52 ± 0.09, and 15.41 ± 0.05, respectively. Insoluble content results were 2.35 ± 0.01, 7.69 ± 0.03, 11.21 ± 0.05, and 14.09 ± 0.07% in WF, MB5, MB10, and MB15. Soluble dietary fiber content was 1.03 ± 0.02, 1.12 ± 0.01, 1.31 ± 0.04, and 1.32 ± 0.05 in WF, MB5, MB10, and MB15 treatments (Table 1). In previous research, Boita et al. (2016) determined the total dietary fiber content in wheat flour with wheat bran incorporation at different levels 6.25%–25% was 3.67% to 12.08%. MB is identified as a good source of several bioactive compounds as well as dietary fiber (Sharma et al., 2016). Therefore, MB‐enriched flour has high total dietary fiber content due to maize bran addition. Dietary fiber helps against cardiovascular diseases, obesity, cancer, and diabetes type II (Cui et al., 2019).

### 3.3 Water‐holding capacity of flour

**FIGURE 1:**
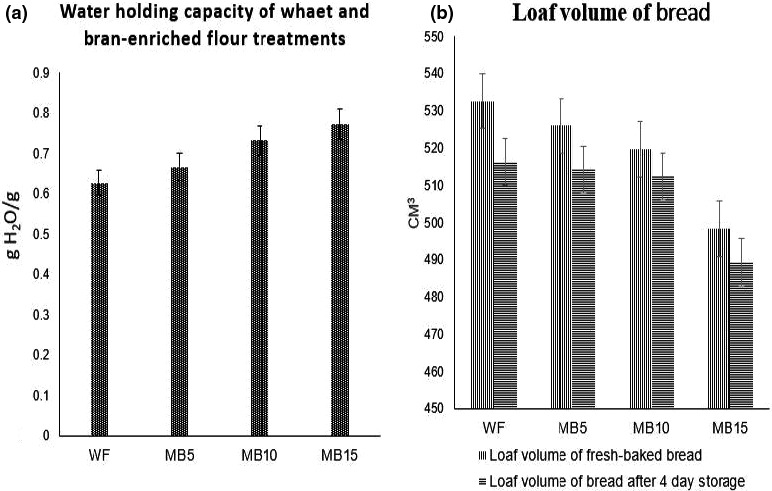
a. Water‐holding capacity of wheat and bran‐enriched flour treatments. b. Loaf volume of bread. WF, Wheat flour; MB5, 5% maize bran + 95% wheat flour; MB10, 10% maize bran + 90% wheat flour; MB15, 15% maize bran + 85% wheat flour

The water‐holding capacity (WHC) is directly associated with the flour and bran content of the sample. The WHC of wheat flour WF and bran containing composite flour MB5, MB10, and MB15 is shown in Figure 1a. WF had a WHC of 0.62 g H_2_O/g. After the addition of maize bran, the WHC of the dough increased up to 0.66 and 0.77 g H_2_O/g. The WHC results were 0.66, 0.73, and 0.77 g H_2_O/g in MB5, MB10, and MB15 treatments. The WHC of MB‐enriched flour treatments was higher than wheat flour as expected because bran bind water due to higher water retention capacity and swelling power. Traynham et al. (2007) indicated that the water‐holding capacity was higher in composite flour. In previous research, Hemdane et al. (2018) explored that incorporation of 20% bran in wheat flour sample showed 0.84 g H_2_O/g water‐holding capacity than wheat flour.

### 3.4 Effect of maize bran on rheological properties of wheat flour dough

#### 3.4.1 Farinographic characteristics

Dough leads to form a wet mass when wheat flour, MB, and water are mixed. The dough is developed during the mixing procedure due to the complex interactions with wheat constituents. Development of the dough starts with the addition of water and continues with mixing process. Water absorption is the amount of water that flour absorbs to maintain the optimal consistency and to produce a better dough for bread preparation. It is the appropriate amount of water you can apply to a dough before the mixture is too sticky. The farinographic results of treatments (WF, MB5, MB10, and MB15) were shown in Table 2. The water absorption results show that WF dough absorbs the lower amount of water 61.98 ± 1.76%, and other maize bran‐enriched flour dough has higher ability of water absorption (66.32 ± 1.9, 68.39 ± 2.35, 68.39 ± 2.35, and 69.69 ± 2.35) in MB5, MB10, and MB15, respectively. These results are not significantly different from each other.

**TABLE 2:** Effect of maize bran on farinographic and mixographic characteristics of wheat flour

Flour Treatments	Farinographic analysis					Mixographic analysis	
	WA (%)	DDT (min)	DS (min)	MTI (%)	*SD* (%)	Peak Height (BU)	Mixing time (min)
WF	61.98 ± 1.76^b^	6.75 ± 0.25^b^	3.98 ± 0.13^a^	63.83 ± 0.02^d^	139.4 ± 0.02^a^	54.23± 0.21^d^	6.23 ± 0.04^a^
MB5	66.32 ± 1.9^a^	7.66 ± 0.38^a^	4.17 ± 0.11^a^	64.07 ± 0.03^c^	138.5 ± 0.02^b^	60.11± 0.13^c^	6.08± 0.03^b^
MB10	68.39 ± 2.35^a^	7.78 ± 0.38^a^	4.32 ± 0.12^a^	64.67 ± 0.01^b^	137.7 ± 0.03^c^	63.19± 0.17^b^	5.88± 0.13^c^
MB15	69.69 ± 2.35^a^	8.25 ± 0.66^a^	4.54 ± 0.6^a^	65.32 ± 0.02^a^	132.3 ± 0.06^d^	67.72± 0.11^a^	5.72± 0.01^d^

Abbreviations: DDT, Dough development time; DS, Dough stability; MB10, 10% maize bran + 90% wheat flour; MB15, 15% maize bran + 85% wheat flour; MB5, 5% maize bran + 95% wheat flour; MTI, Mixing tolerance index; *SD*, Softness of dough; WA, Water absorption; WF, Wheat flour.

Maize bran had higher swelling strength and water retaining potential than wheat flour, and improved water absorption could minimize the negative impact of adding maize bran on the gluten network development and the quality of bread. The hydration mechanism is accomplished by forming hydrogen bonds and hydrophilic interactions with the water molecules in protein and starch molecules. Dough mixing is a procedure in which flour and water are stirred until gluten is formed as a consequence of the increased interaction among dispersed and hydrated gluten‐forming proteins. The dough development period was high in MB15 8.25 ± 0.6 min followed by WF, MB5, and MB10 were 6.75 ± 0.25, 7.66 ± 0.38, and 7.58 ± 0.38b, respectively. The development time for dough WF (6.75 ± 0.25 min) was lower than the bran‐enriched maize dough, and this could be due to increased maize bran surface area. In the process of developing dough especially bread dough, the objective was to introduce some physical changes in the dough properties, to enhance its ability to retain the CO_2_ gas released during the fermentation. Dough stability is the stage where the gluten breaks down and the dough over mixes. In all the dough systems, mixing is a critical step, that is influenced by the speed of the mixer, dough temperature, water absorption of the flour, and shortening amount in the dough recipe. The dough stability time results were 3.98 ± 0.13, 4.17 ± 0.11, 4.32 ± 0.12, and 4.54 ± 0.6 in WF, MB5, MB10, and MB15 treatments, respectively. Mean values showed that the addition of maize bran caused a nonsignificant increase in dough stability time. This means that bran has a high potential to absorb water, and this makes the dough less stable. The mean values for the effect of maize bran on the mixing tolerance index of wheat flour and increasing pattern for mixing time were observed by applying treatments, that is, WF (control), MB5, MB10, and MB15. The mixing tolerance index was 63.83 ± 0.02, 64.07 ± 0.03, 64.67 ± 0.01, and 65.32 ± 0.02% in WF, MB5, MB10, and MB15, respectively, in wheat flour. It is obvious from these results that the mixing tolerance index of wheat flour is increased with the addition of MB.

Furthermore, a decreasing trend was observed in the softness of wheat flour dough by adding maize bran with varying concentrations. The results revealed that the softness of dough was 139.4 ± 0.02, 138.5 ± 0.02, 137.7 ± 0.03, and 132.3 ± 0.06% in WF (control), MB5, MB10, and MB15, respectively.

#### 3.4.2 Mixographic characteristics

Results regarding means values of the peak height variation of wheat flour owing to maize bran addition showed a significant increase in peak height. In MB15 treatment, the highest peak height (67.72 ± 0.11 BU) was observed in flour with 15% maize bran. The peak height results were significantly increased with the addition of maize bran (Table 2).

Mean values regarding the effect of maize bran on the mixing time of wheat flour showed a significant decrease in mixing time by applying treatments (5%, 10% and 15% maize bran) as shown in Table 3. In WF, MB5, MB10, and MB15 treatments, mixing time was 6.23 ± 0.04, 6.08 ± 0.03, 5.88 ± 0.13, and 5.72 ± 0.01 min, respectively. The results regarding mean values revealed that the highest mixing time (6.23 ± 0.04 min) was observed in WF control treatment, whereas the lowest mixing time (5.72 ± 0.01 min) was exhibited by flour with MB15 (15% maize bran).

**TABLE 3:** Mean values of moisture content, hardness and fracturability of bread

Bread treatments	Moisture content (%)	Hardness (*N*)	Fracturability (*N*)
WF	34.54 ± 0.05^a^	22.02 ± 0.02^a^	1.02 ± 0.03^b^
MB5	35.73 ± 0.02^a^	22.19 ± 0.03^a^	0.96 ± 0.01^ab^
MB10	37.2 ± 0.09^ab^	22.34 ± 0.03^a^	0.95 ± 0.02^a^
MB15	38.09 ± 0.11^b^	22.91 ± 0.04^a^	0.93 ± 0.01^a^

Note

Abbreviations: MB10, 10% maize bran + 90% wheat flour; MB15, 15% maize bran + 85% wheat flour; MB5, 5% maize bran + 95% wheat flour; WF, Wheat flour.

#### 3.4.3 Comparison of dough height and bread height

**FIGURE 2:**
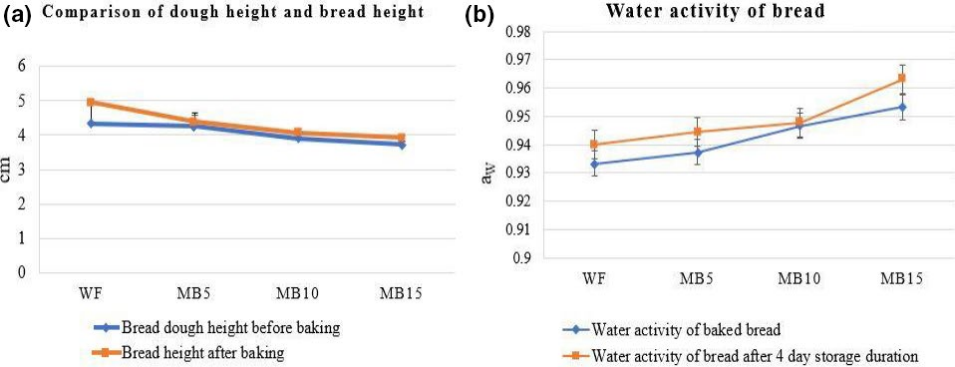
a. Comparison of dough height and bread height. b. Water activity of bread. WF, Wheat flour; MB5, 5% maize bran + 95% wheat flour; MB10, 10% maize bran + 90% wheat flour; MB15, 15% maize bran + 85% wheat flour

The bread dough height and after baking bread height mean values result were depicted in Figure 2a. As expected, the WF sample showed highest value for bread dough and after baking bread height. Bread height varies inversely with maize bran addition to bread dough flour. This could be due to exceeding the absorption of water, which reduces the dough's gas‐free density. Present findings indicated that bran water‐binding ability under stressed conditions (oven) induced a reduction in gas retention with maize bran‐enriched dough. The bread dough height values after proofing of WF, MB5, MB10, and MB15 were 4.32 ± 0.04, 4.26 ± 0.01, 3.9 ± 0.02, and 3.72 cm, respectively.

After baking, the results for height after baking showed the maximum values. The bread height is greater than the dough height due to the baking fermentation process. Heat speed up the fermentation mechanism which illustrates when bread keeps rising in the first few minutes of baking in the oven. The bread height value was observed in samples WF, MB5, MB10, and MB15 followed by 4.96 ± 0.7, 4.38 ± 0.2, 4.06 ± 0.1, and 3.91 ± 0.06 cm, respectively. The particle of the maize bran causes the gas cell more resilient and sensitive to breakage, and that they develop a physical barrier around the gas cells, pressuring them to expand in a particular dimension (Jacobs et al., 2016). When the gas cells expand, the bran particles align with the gas cells, developing a physical wall that may hinder proper expansion of the gas cells.

#### 3.4.4 Bread loaf volume

The mean values of bread volume have been shown in Figure 1b. It is visible that volume was significantly affected by different treatments and storage periods. Likewise, a significant difference between treatments and day interaction was noticed. The size of the bran particle has a certain impact on the volume of the bread loaf. This has been reported by many scientists that the bran particle size is associated with gas retention and the fine volume of the bread.

The mean values for loaf volume (Figure 1 b) suggested that values decreased in the volume of bread by the various rates of bran treatment while a decreasing trend in all treatments, volume of bread was recorded during the storage period. In all treatments (WF, MB5, MB10, and MB15), result values were volume 532.67 ± 10.59, 526 ± 7.21, 519.66 ± 8.08, and 498.33 ± 5.13 cm^3^, respectively, at 0‐day (freshly baked bread after cooling at room temperature). The volume of bread decreased after 4‐day duration, and values were 516.33 ± 8.02, 514.32 ± 4.58, 512.33 ± 13.79, and 489.33 ± 15.37 cm^3^ in WF, MB5, MB10, and MB15 treatments, respectively. WF treatment bread volume was high than other bran‐enriched treatments. The incorporation of bran to flour normally results in undesirable effects on the properties of the bread dough like. Schmiele et al. (2012) reported that a reduction in the specific volume from 4.4 down to 1.8 cm^3^/g when wheat flour was enriched by wheat bran up to 40%. Ultimately, introducing bran to flour contributes to undesirable effects on the properties of dough, the volume of bread loaf, texture, color, and taste.

#### 3.4.5 Moisture content of bread

The moisture level of various food items is one of the most essential and widely measured properties. It is calculated for a multitude of reasons, such as legal and labeling criteria, economic importance, food quality, increased efficiency, and safety of storage. The mean values of moisture content in bread are described in Table 3. Data relating to moisture contents of different bread treatments WF, MB5, MB10, and MB15 progressive increase in this attribute with the gradual increase of maize bran level. The values of treatments WF, MB5, MB10, and MB15 bread moisture content were 34.54 ± 0.05, 35.73 ± 0.02, 37.2 ± 0.09, and 38.09 ± 0.11%, respectively, and may be related to the high ability of bran to absorb water. The water absorption property of dough improved with the addition of maize bran level, that is why the moisture content of bread was increased with high bran concentration. These results are equivalent to Pauline et al. (2020) reported that wheat bran‐enriched bread has 30% moisture.

#### 3.4.6 Bread hardness and fracturability

The bran‐enriched bread indicated higher water content but often higher hardness, indicating that the moisture content is not the only aspect affecting the hardness and may not have been sufficiently imperative to overcome the negative influence of bran fractions on texture (Boita et al., 2016). Chen et al. (2011) observed that the smaller size of the bran particles resulted in significantly greater hardness. Other factors such as water molecular, redistribution, water dynamics, and gluten network changes (i.e., loss of plasticity) are reported to contribute to the increased hardness (Curti et al., 2015).

Hardness is a substantial qualitative parameter that affects the end‐use of given maize bran. The results regarding statistical analysis for bread hardness of different treatments of bread prepared at different levels of maize bran. It is manifest from the statistical analysis that the hardness of the bread was affected significantly by different levels of maize bran. Results indicated that the hardness of bread slightly increased significantly due to the increase in levels of maize bran. The bread treatments WF, MB5, MB10, and MB15 hardness result values were 22.02 ± 0.02 22.19 ± 0.03, 22.34 ± 0.03, and 22.91 ± 0.04N, respectively. Bread with higher moisture, specific volume, water activity, and more hardness was also produced by the presence of the bran. In previous research, Curti et al. (2013) indicated that the incorporation of bran fractions with different particle sizes did not significantly affect the hardness of the samples.

Fracturability is the ease with which a sample crumbles cracks or shatters also called brittleness. Brittle solid food exhibited fracturability and possessed low cohesiveness and more hardness. Change in the fracture intensities results in low moisture and deformation of the bread. Means values regarding bread fracturability are depicted in Table 3. The values for fracturability of bread were 1.02 ± 0.05, 0.96 ± 0.01, 0.95 ± 0.02, and 0.93 ± 0.01 N in WF, MB5, MB10, and MB15 treatments, respectively.

#### 3.4.7 Water activity of bread

The water activity of bread indicates the lower limit of water availability for microbial growth. It is critical to regulating water activity to preserve the chemical stability of foods. Water activity plays a significant role in the physical properties of foods including texture and shelf life. Labuza et al. (1972) indicated that preventing microbial spoilage is done by reducing water activity below 0.7. Even if the food would not spoil from microorganisms, there may still be other deteriorating reactions.

The mean values regarding the water activity of different bread prepared with wheat flour with the addition of MB are given in Figure 2b. The means results for the water activity showed an increasing trend with the intervals of storage (0 and 4 days) in the bread treatments WF, MB5, MB10, and MB15. The water activity of bread in 0‐day results values was 0.933 ± 0.015, 0.937 ± 0.01, 0.946 ± 0.01, and 0.953 ± 0.01 in WF, MB5, MB10, and MB15 treatments, respectively. The treatments maintained the same results and, in some cases, decreased. It is noticeable from the results that a gradual increase in the water activity was observed with the regular increase of MB in the different treatments. At 0‐day analysis, there was a decreasing trend in the water activity with various levels (MB5, MB10, and MB15) in different treatments. After 4‐days storage, an increasing trend was exhibited 4 days of storage interval which showed that water activity increased in some cases, WF, MB5, MB10, and MB15 from 0.94 ± 0.01, 0.944 ± 0.02, 0.947 ± 0.02, and 0.963 ± 0.005 respectively, after 4 days. A similar movement was detected throughout the research that the water activity was lower with the addition of MB. The influence of storage on water activity showed a gradual increase in water activity values for each trait but, meanwhile, the effect of the MB addition was also noticeable in the reduction of water activity of baked bread as a function of various levels.

#### 3.4.8 Sensory evaluation

Sensory evaluation of WF, MB5, MB10, and MB15 samples is presented in Table 4. The panelist scored the four bread on color and appearance of bread between “like moderately” and “like very much.” Since the panelists were asked to score the bread individually and not compare between them, there were no statistically significant differences scores for color and appearance. Texture and taste scores for the four bread were between “like slightly” and “like moderately.” Taste scores were low as no additional flavoring was added. Maize bran addition at 5%, 10%, and 15% level in bread did not significantly affect the overall acceptability scores. Based on the data in this study, it can be recommended that up to 15% of maize bran can be used in bread and bread‐like products. Texture sensory “like slightly” by adding maize in the formulation, thus improving the overall acceptability of high‐fiber bread containing finely milled MB.

**TABLE 4:** Sensorial properties of bran‐enriched bread

Parameter	WF Control	Bread with maize bran addition		
		MB5	MB10	MB15
Appearance	8.41^a^	8.26^a^	8.35^a^	8.02^a^
Color	7.98^a^	7.72^a^	7.58^a^	7.11^a^
Taste	7.63^a^	7.58^a^	7.61^a^	7.47^a^
Flavor	7.71^a^	7.62^a^	7.75^a^	7.03^a^
Texture	7.11^a^	6.87^a^	6.83^a^	6.15^a^
Overall acceptability	7.71^a^	7.56^a^	7.58^a^	7.10^a^

^a^ Means in the same column with the same superscript are not significantly different (*p* = .05) according to Duncan's Multiple Range Test.

## 4 CONCLUSION

Maize bran is rich in nutritional as well as bioactive profile than bran of other cereals. It comprised of high dietary fiber content that showed positive effect on nutritional and rheological characteristics of wheat flour. Furthermore, bread prepared with the addition of maize bran at different levels 5%, 10%, and 15% in flour improved the rheological and textural and physicochemical characteristics of bread. Moreover, MB‐enriched bread (MB10) explicated high score of sensorial overall acceptability.

## CONFLICT OF INTEREST

The authors declare no conflict of interest.

5

**FIGURE 3:**
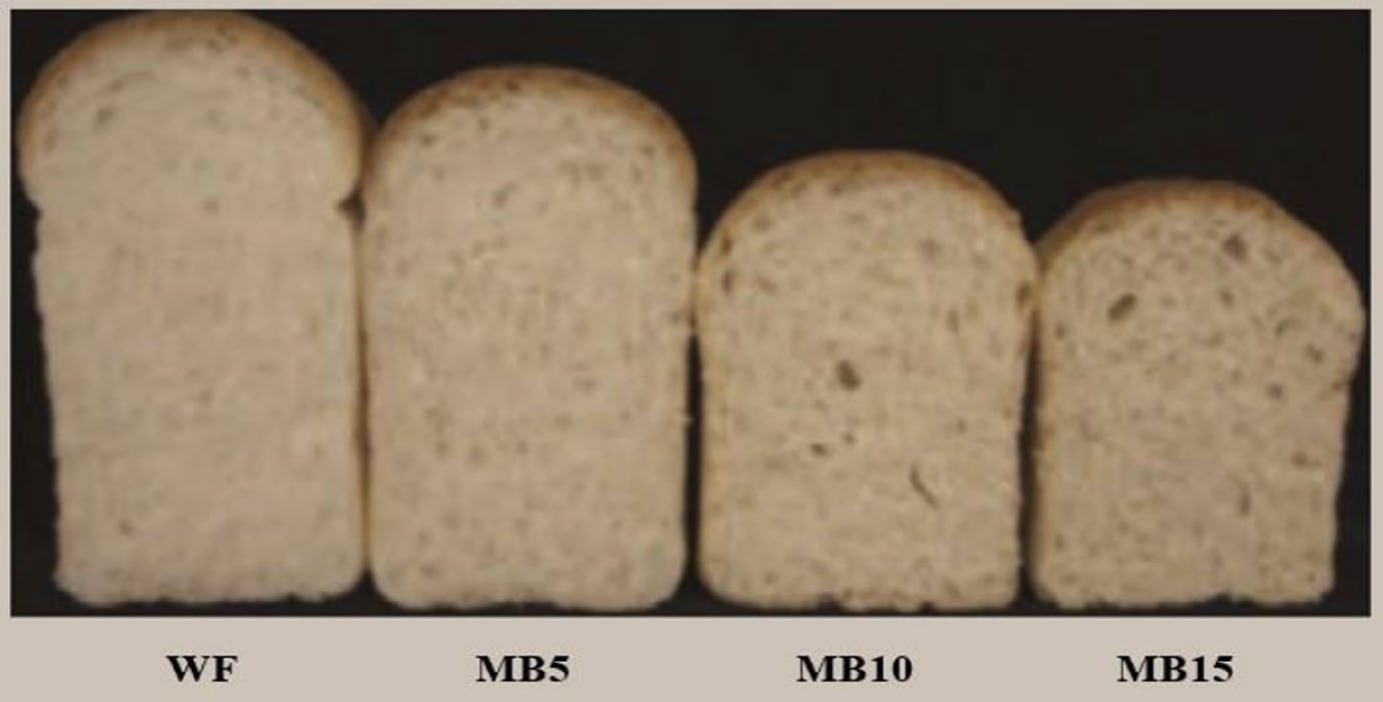
Preparation of bread. WF, Wheat flour; MB5, 5% maize bran + 95% wheat flour; MB10, 10% maize bran + 90% wheat flour; MB15, 15% maize bran + 85% wheat flour
